# Expression Profiling of the Aluminum-Activated Malate Transporter (ALMT) Gene Family in Pumpkin in Response to Aluminum Stress and Exogenous Polyamines

**DOI:** 10.3390/plants14243745

**Published:** 2025-12-09

**Authors:** Xinqi Guo, Mingshan Wang, Qiang Chen, Ying Zhang, Chong Zhang

**Affiliations:** College of Life Science, Shenyang Normal University, 253 Huanghe North Street, Huanggu District, Shenyang 110034, China; a13700189259@126.com (X.G.); 13304582344@163.com (M.W.); chq521900@126.com (Q.C.)

**Keywords:** aluminum stress, pumpkin, polyamines, *ALMT* gene family, gene expression

## Abstract

Aluminum (Al) toxicity is a key constraint on plant growth in acidic soils. To counteract Al stress, plants secrete organic acids such as malate from their roots to chelate Al^3+^, a process facilitated by Al-activated malate transporters (ALMTs). In this study, we identified 15 *ALMT* genes in the pumpkin (Cucurbita moschata) genome, which were phylogenetically classified into four subclades. Expression analysis revealed that several ALMTs, including *CmaALMT5*, *CmaALMT6*, and *CmaALMT12*, were upregulated in response to increasing Al concentrations. Exogenous application of polyamines (spermine, spermidine, and putrescine) alleviated Al-induced root growth inhibition, correlating with enhanced malate secretion. Notably, each polyamine differentially regulated specific ALMT genes: spermidine elevated the expression of *CmaALMT1*, *CmaALMT6*, *CmaALMT13*, and *CmaALMT15*; spermine induced *CmaALMT1*, *CmaALMT2*, *CmaALMT3*, *CmaALMT11*, and *CmaALMT14*; while putrescine significantly upregulated *CmaALMT1*, *CmaALMT3*, and *CmaALMT4*. These results suggest that polyamines may enhance Al tolerance in pumpkin through gene-specific transcriptional regulation of the ALMT family and promotion of root malate secretion, though further evidence is required.

## 1. Introduction

In acidic soils (pH < 5.5), elevated concentrations of aluminum ions (Al^3+^) cause severe toxicity in plants, primarily manifesting as root growth inhibition, disruption of cellular structure and function, and systemic physiological disorders. Aluminum toxicity initially affects the root apical meristematic and elongation zones, where it disrupts microtubule organization and cytoskeletal stability, interferes with cell cycle progression, and consequently leads to restricted root elongation, abnormal morphology, and limited lateral root development. This damage subsequently impairs the water and nutrient absorption capacities of plants. Beyond this direct physical harm, Al^3+^ further exacerbates nutrient deprivation by forming insoluble aluminum (Al)-PO_4_ precipitates with phosphate in the rhizosphere, thereby intensifying phosphorus deficiency [1].This condition also competitively inhibits the absorption of essential elements, such as calcium (Ca^2+^) and magnesium (Mg^2+^), ultimately resulting in reduced shoot growth, decreased photosynthetic efficiency, and significant crop yield reduction [2]. Aluminum stress induces a burst of Reactive Oxygen Species (ROS) [3], triggering membrane lipid peroxidation and malondialdehyde (MDA) accumulation, which compromises cell membrane integrity. In sensitive crops, such as cucumber (*Cucumis sativus*), this process manifests as a decreased root vigor and leaf chlorosis [4]. For example, aluminum stress severely inhibits root development in sorghum (*Sorghum bicolor*) [5] through aluminum accumulation in the root elongation zone, extensive callose deposition [6], and impaired cell membrane integrity. In rice (*Oryza sativa*), the effect of Al on root growth is concentration-dependent; low concentrations promote linear elongation, whereas high concentrations inhibit growth and induce ROS accumulation and cortical tissue damage [7,8].

Plants have developed two primary mechanisms to manage Al stress: external exclusion and internal tolerance [9,10,11]. The Al exclusion mechanism depends on the rapid secretion of organic acids, such as citrate and malate, from roots. These organic acids chelate Al^3+^ in the rhizosphere, forming non-toxic complexes (e.g., Al-citrate) that prevent cellular metal entry. The transcription factor *STOP1* [12] regulates this process by activating downstream organic acid transporter gene expression following its phosphorylation and nuclear localization. The Al-activated malate transporter (ALMT) gene family plays a crucial role in the plant response to Al stress by mediating malate secretion. ALMT proteins facilitate malate efflux and form stable complexes with aluminum ions to mitigate Al toxicity. Studies have shown that Al-tolerant genotypes (*Lens culinaris*) demonstrate significantly higher organic acid secretion than sensitive genotypes [13]. In soybean (*Glycine max*), the *GmALMT1* gene encodes a malate transporter protein, whose function and expression are regulated by extracellular pH, with synergistic modulation by pH, Al, and phosphorus, enhancing plant adaptation to acidic soil through increased malate efflux into the rhizosphere [14]. Additionally, several other ALMT family members, including *TaALMT1* in wheat (*Triticum aestivum*) [15], *BnALMT1* and *BnALMT2* in rapeseed (*Brassica napus*) [16], and *AtALMT1* in *Arabidopsis thaliana* [17], confer Al tolerance through an Al-activated malate efflux mechanism. The conserved function of the ALMT family in crop Al tolerance has been further validated in potato (*Solanum tuberosum*), where *StALMT6* and *StALMT10* showed significantly upregulated expression under Al treatment, and their overexpression resulted in enhanced Al resistance and malate secretion capacity [18]. The ALMT gene family has been systematically characterized in various plant species. For example, 34 GmALMT members have been identified in the soybean genome, with *GmALMT5* confirmed as a plasma membrane protein mediating root malate efflux [19]. In sugarcane (*Saccharum officinarum*), 11 ALMT members have been identified, with six involved in the aluminum toxicity response [20]. In Chinese white pear (*Pyrus bretschneideri*) [21] and apple (*Malus domestica*) [22], the ALMT family plays crucial roles in physiological processes, including malate accumulation and organic acid efflux.

Recently, the role of polyamines (PAs) in plant response to aluminum (Al) stress has received increased attention [23,24]. Plants maintain polyamine homeostasis by maintaining a dynamic balance between polyamine synthesis and catabolism. PA synthesis begins with arginine and ornithine, which are catalyzed by Arginine Decarboxylase (ADC) [25] and Ornithine Decarboxylase (ODC) [26], respectively, to produce putrescine (Put), which is subsequently converted to spermidine (Spd) and spermine (Spm) through aminopropyl addition [27]. PA catabolism occurs primarily through oxidative deamination reactions catalyzed by Polyamine Oxidase (PAO) and Diamine Oxidase (DAO) [28,29]. Al stress disrupts PA homeostasis by altering the content and ratio of PA components, thereby affecting Al tolerance in plants. Polyamines mitigate Al toxicity through several mechanisms: First, they modify cell wall properties to reduce Al accumulation. Putrescine decreases cell wall pectin content and enhances methylesterification, thereby reducing Al-binding sites [30,31,32,33]. Research has demonstrated that exogenous Put treatment reduces Al content in wheat root tip cell walls, significantly alleviating root elongation inhibition [34]. Second, ROS balance is regulated to reduce oxidative damage. Polyamines directly scavenge ROS and suppress PAO and NADPH oxidase activities, thereby reducing H_2_O_2_ production [35]. The H_2_O_2_-Spd signaling pathway triggered by PAO enhances tomato (*Solanum lycopersicum*) tolerance to salt-alkali stress [36]. Third, it interacts with ethylene signaling to modulate the stress response. Polyamines reduce growth inhibition by suppressing the activity of ACS, which is a key enzyme in ethylene synthesis, thereby limiting Al-induced ethylene production [37]. Studies on silicon treatment have shown that activating PA synthesis while suppressing ethylene synthesis in peach trees limits gummosis progression [38]. Species-specific mechanisms exist: Al-tolerant wheat genotypes increase Put content via the ADC pathway [39]; in rice, ArPK affects Put synthesis by regulating ODC activity, where ODC overexpression reduces Al tolerance [40]; and tea tree (*Camellia sinensis*) increases Put accumulation 37-fold through arginine metabolism to chelate aluminum and maintain cellular homeostasis [41].Moreover, PAs degradation intermediates can be converted into γ-aminobutyric acid (GABA). Specifically, putrescine and spermidine are converted into Δ^1^-pyrroline by the action of diamine oxidase and polyamine oxidase, respectively, and Δ^1^-pyrroline is then catalyzed by pyrroline dehydrogenase to form GABA [42,43]. Further research demonstrated that the activity of *TaALMT1* in barley is regulated by GABA: under acidic conditions with Al^3+^, aluminum-tolerant wheat roots exhibit low GABA levels and enhanced malate efflux, while in the absence of Al^3+^, GABA accumulates and malate efflux is inhibited [44]. Exogenous GABA treatment also suppresses malate efflux and reduces aluminum tolerance in plants. These findings suggest that polyamines may regulate ALMT function through their degradation product GABA, thereby influencing plant aluminum tolerance. However, whether the polyamine regulation of ALMT pathway constitutes a key mechanism in plant aluminum stress response still requires further experimental evidence.

‘Beibei’ pumpkin (*Cucurbita moschata ‘BeBe’*), an annual cucurbit crop, is widely cultivated and highly valued by consumers. However, Al stress significantly inhibits root growth, resulting in a decreased yield [45,46]. The identification of the pumpkin *ALMT* gene family and its expression regulation under Al stress remains unreported. Additionally, the role of PAs as signaling molecules in modulating ALMT-mediated Al tolerance mechanisms in pumpkins requires further clarification. This study aimed to identify the pumpkin *ALMT* gene family members at the genome-wide level, examine their expression patterns under Al stress and polyamine treatment, and elucidate the synergistic mechanism between polyamine metabolism and ALMT-mediated organic acid secretion in pumpkin Al tolerance. These findings provide a theoretical foundation for understanding the molecular mechanisms underlying Al tolerance in pumpkins.

## 2. Results

### 2.1. Identification of the Pumpkin ALMT Gene Family

Pumpkin genome analysis revealed 15 ALMT family members, designated as *CmaALMT1* to *CmaALMT15* (Table 1). These genes demonstrated significant diversity in their molecular characteristics, including amino acid (aa) composition, molecular weight (MW), and theoretical isoelectric point (pI). *CmaALMT13* possessed the largest protein structure with 1215 aa and 136.72 kDa, while *CmaALMT8* exhibited the smallest with 328 aa and 36.89 kDa. Theoretical pI analysis revealed that most CmaALMT proteins were weakly acidic to neutral, with values between 5.84 and 8.99. Four members, *CmaALMT8*, *CmaALMT11*, *CmaALMT14*, and *CmaALMT15*- displayed pI values exceeding 8.5, thus categorizing them as basic proteins. Transmembrane domain (TMD) analysis indicated that all members contained 5–11 transmembrane helices. *CmaALMT7* exhibited the highest number of TMDs (11), whereas *CmaALMT2*, *CmaALMT10*, *CmaALMT13*, *CmaALMT14*, and *CmaALMT15* each contained five TMDs. Subcellular localization predictions indicated that *CmaALMT7* was targeted to the nucleus (Nucl), whereas the remaining 14 members were predicted to localize to the plasma membrane (Plas), suggesting a potential role in malate transmembrane transport.

**Figure 1:**
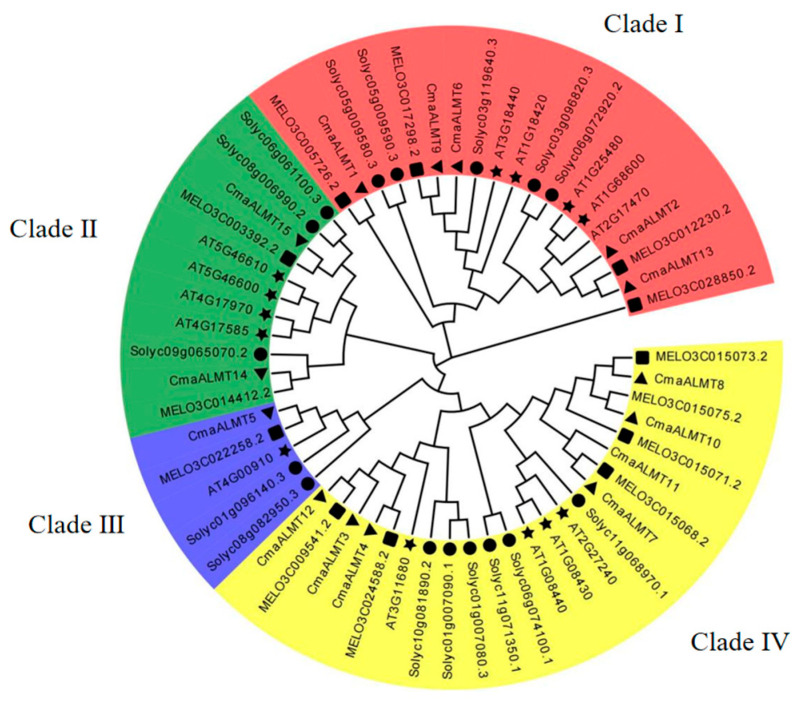
Phylogenetic analysis of *ALMT* gene family members. The evolutionary tree was constructed using the ALMT protein sequences from *Cucurbita moschata* (*Cma*), *Solanum lycopersicum* (*Sl*), *Cucumis melo* (*ME*), and *Arabidopsis thaliana* (*At*). The tree is divided into four distinct clades (I–IV), indicated by red, green, blue, and yellow background shades, respectively. The scale bar represents the number of amino acid substitutions per site.

Evolutionary relationships within the pumpkin ALMT gene family were examined by aligning the CmaALMT protein sequences with homologous ALMT sequences from *Cucurbita moschata* (*Cma*), *Solanum lycopersicum* (*Sl*), *Cucumis melo* (*ME*), and *Arabidopsis thaliana* (*At*), followed by phylogenetic tree construction (Figure 1). The analysis revealed four major clades (I, II, III, and IV). Clade I, the most thoroughly characterized subgroup, primarily functions in Al-activated malate efflux, which is essential for plant Al tolerance. This clade encompassed five pumpkin, five *Arabidopsis*, five tomatoes, and four melon homologs. Clade II members have diverse functions, including broad-spectrum anion channels, stomatal movement, and water balance regulation. This subgroup comprises two pumpkin, four *Arabidopsis*, three tomato, and two melon homologs. Clade III represented a highly conserved basal subgroup with minimal members (five in total), including one member each from pumpkin, melon, and *Arabidopsis*, and two from tomato. Its limited redundancy and cross-species conservation suggests that it plays a fundamental role in maintaining basic cellular physiological functions. Clade IV, which contained 23 members, exhibited the most substantial expansion in the pumpkin ALMT family. Pumpkin contributed seven members to this clade, representing the highest species contribution, along with six members each from melon and tomato, and four from Arabidopsis. This distribution indicated a significant lineage-specific expansion of pumpkin *ALMT* genes within Clade IV.

### 2.2. Gene Structure and Promoter Analysis

**Figure 2:**
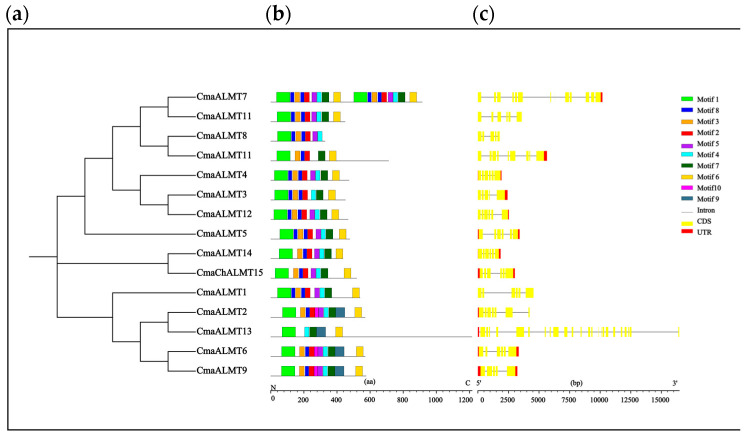
Phylogenetic Analysis, Conserved Motifs, and Gene Structure of *CmaALMT* Gene Family Members in Pumpkin. (**a**) Phylogenetic tree constructed based on the protein sequences of the *CmaALMT* family members, showing their evolutionary relationships and clustering into distinct clades. (**b**) Analysis of conserved motifs (Motifs) in the *CmaALMT* gene family members. Different colors and numbers represent distinct motifs. (**c**) Exon–intron structure diagram of the *CmaALMT* genes. Green, yellow, and grey boxes represent the Untranslated Regions (UTRs), Coding Sequences (CDs), and introns, respectively.

Structural analysis of the 15 identified pumpkin *ALMT* genes revealed that all members contained coding sequences (CDs), untranslated regions (UTRs), and introns with varying compositions and distributions(Figure 2a). UTR analysis showed that most genes (*CmaALMT7*, *CmaALMT8*) contain both 5′ and 3′ UTRs, while some members possessed only the 3′ UTR(Figure 2b). The CDs demonstrated high conservation among different members, aligning with their essential roles in fundamental biological functions, such as aluminum ion transport(Figure 2c). The number of introns varied notably between genes; for example, genes such as *CmaALMT3* contained 2–3 introns, whereas others, such as *CmaALMT12*, possessed 4–5 introns. This structural variation may influence the efficiency and functional specificity of gene transcription. The analysis identified 10 distinct conserved motifs. Motifs 1, 2, and 3 formed the core elements present in all CmaALMT proteins, with motif 1 maintaining a fixed position across all members, indicating its essential role in basic transcriptional regulation. Some motifs showed specific distribution patterns: motifs 8 and 5 appeared exclusively in select members, such as *CmaALMT7*; motifs 4 and 7 frequently occurred as adjacent pairs in multiple members; and motifs 6, 10, and 9 were unique to individual genes, such as *CmaALMT14*. These specific motifs may correlate with the stress response functions of the genes, providing structural insights into the role of *CmaALMT* genes in Al stress and polyamine regulation mechanisms.

**Figure 3:**
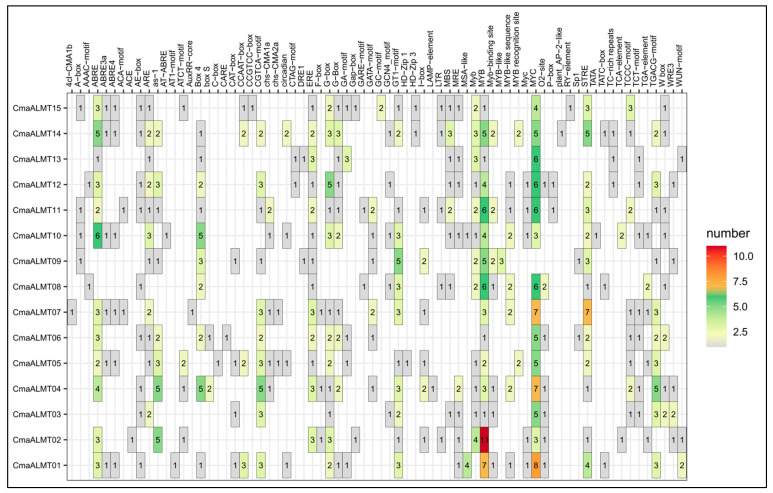
Heatmap Analysis of Cis-Acting Elements in the Promoter Regions of *CmaALMT* Gene Family Members in Pumpkin. The vertical axis represents the different *CmaALMT* gene members, and the horizontal axis represents the types of cis-acting elements located within the promoter regions (2000 bp upstream of the transcriptional start site). The color intensity and numerical values within the heatmap indicate the quantity of a specific element present in each gene.

Analysis of the promoter regions (2000 bp upstream of the transcriptional start site) of the 15 *CmaALMT* genes (Figure 3) identified over 80 types of cis-acting elements encompassing multiple functional categories, including hormone responsiveness, stress response, and general transcriptional regulation. This composition establishes a molecular foundation for the complex transcriptional regulation of *CmaALMT* genes. Hormone-responsive elements were particularly abundant, including the ABRE element involved in the abscisic acid (ABA) signaling pathway and the TGACG motif associated with the jasmonic acid response. The promoter region also contained numerous elements linked to abiotic stress responses, such as STRE, which is related to oxidative stress. Additionally, the binding sites for transcription factors, including MYB and MYC, are widely distributed. The promoter regions of different *CmaALMT* genes exhibited significant variation in element type and number, potentially explaining their functional differentiation. For example, STRE was significantly enriched in the promoters of *CmaALMT02* and *CmaALMT08*, suggesting a specific role in the oxidative stress response, whereas *CmaALMT01* and *CmaALMT02* contained numerous regulatory elements (highlighted in orange in Figure 3), indicating regulation by a more complex transcriptional network. These findings suggest that Al stress activates specific *CmaALMT* genes through the corresponding *cis*-elements via oxidative stress signals and hormone pathways induced by Al. Furthermore, polyamines, which are crucial signaling molecules, may indirectly influence these promoter elements by regulating MYB/MYC transcription factor activity or by mediating hormone signal transduction, ultimately controlling the differential expression of *CmaALMT* genes under stress.

### 2.3. Expression Analysis of ALMT Genes Under Al Stress

**Figure 4:**
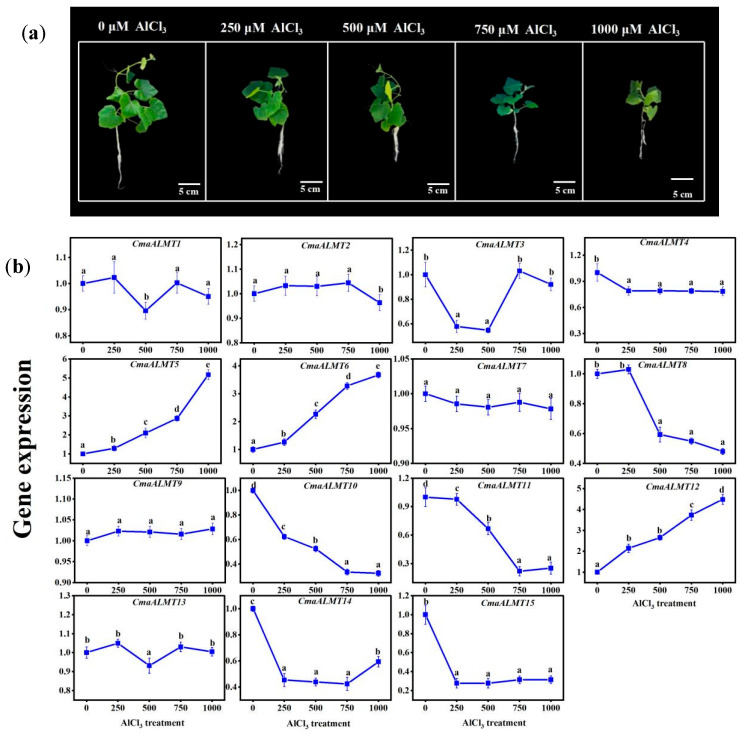
Phenotype of Pumpkin Seedling Roots (**a**) and Expression Levels of *ALMT* Gene Family Members (**b**) Under Different Aluminum Concentrations. Each data point represents the mean ± S.E. (*n* = 3). Different lowercase letters on the bar indicated significant difference (*p* < 0.05).

To examine the effect of Al stress on pumpkin root growth and the expression patterns of its ALMT gene family members, we implemented treatments using varying Al^3+^ concentrations. Phenotypic analysis (Figure 4A) demonstrated that Al stress affected root growth in a concentration-dependent manner. Low-concentration Al treatments (250–500 μM) inhibited primary root elongation while promoting lateral root development. In contrast, high-concentration Al stress (750–1000 μM) significantly suppressed the growth of both primary and lateral roots, with root damage intensifying as Al^3+^ concentration increased. Expression analysis of the *CmaALMT* genes using qRT-PCR (Figure 4B) revealed diverse responses to Al stress. The expression levels of *CmaALMT5*, *CmaALMT6*, and *CmaALMT12* showed a consistent upward trend with increasing Al concentration, maintaining elevated levels in the high-concentration range (750–1000 μM), exhibiting typical Al-inducible expression characteristics. Conversely, the expression of *CmaALMT10* and *CmaALMT11* decreased continuously as Al concentration increased, reaching their lowest levels at 1000 μM. Some genes displayed concentration-specific fluctuating response patterns: *CmaALMT3* expressed highly under both Al-free and high-Al (750 μM) conditions but was suppressed in the medium-to-low concentration range (250–500 μM). *CmaALMT8* exhibited an expression peak at 250 μM before declining rapidly. *CmaALMT14* showed high basal expression under Al-free conditions, was suppressed under medium-concentration Al stress, but demonstrated partial expression recovery at 1000 μM. Additionally, genes, including *CmaALMT2*, *CmaALMT7*, and *CmaALMT9,* maintained relatively stable expression levels across all Al concentrations without significant regulation. These genes may not directly participate in the Al stress response but rather contribute to basic metabolism or cellular homeostasis maintenance under non-abiotic stress conditions.

### 2.4. Effects of Exogenous Polyamines on Root Phenotype and Malate Content in Pumpkin Seedlings Under Aluminum Stress

**Figure 5:**
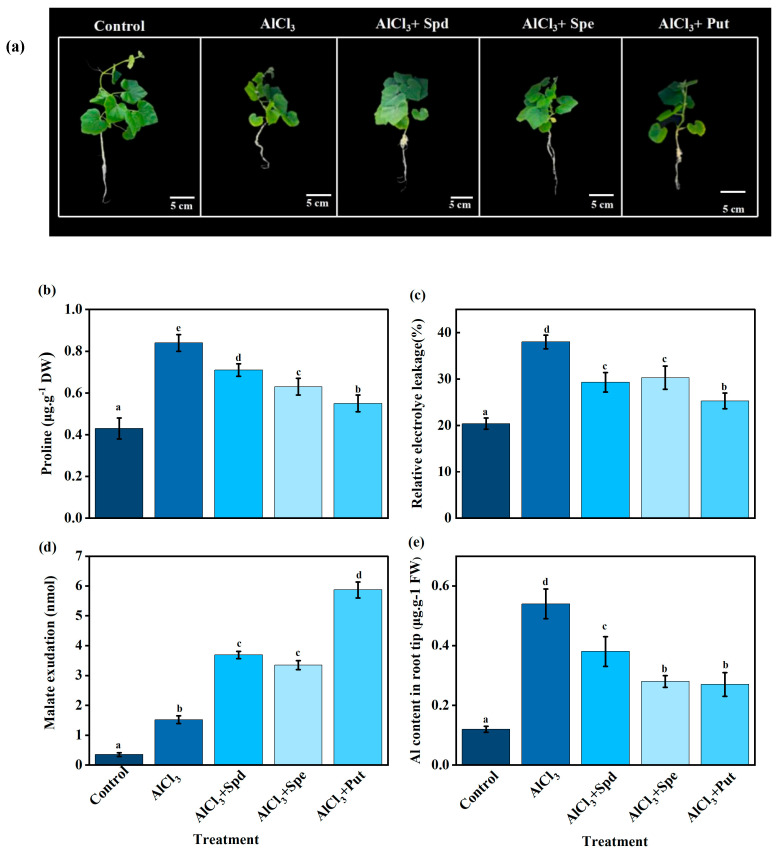
Effects of Different Polyamines on the Root Phenotype (**a**), Proline Content (**b**), Relative electrolyte leakage (**c**), Malate exudation Content (**d**), and Al content (**e**) of Pumpkin Seedlings Under Aluminum Stress. Each data point represents the mean ± S.E. (*n* = 3). Different lowercase letters on the bar indicated significant difference (*p* < 0.05).

To examine the mitigating effects of exogenous PAs against Al stress, we analyzed the effects of spermine (Spm), spermidine (Spd), and putrescine (Put) on pumpkin seedling root growth. The phenotypic results indicated that singular Al stress (1000 μM) significantly inhibited root growth compared to the control. Spd and Put had the most pronounced effects on lateral root development (Figure 5a). PAs significantly reduced the increase in proline content and root relative electrical conductivity under Al stress (Figure 5b and c). Furthermore, the external exclusion of Al toxicity through malate secretion represents a key mechanism of Al tolerance. We measured the levels of malic acid in the apical roots. As shown in Figure 5d, different types of polyamines increased the content of malic acid in the root under aluminum stress to varying degrees, among which putrescine increased the highest level, which was approximately 16.77 times higher than that under Al stress. Polyamine treatment significantly inhibited aluminum content in plant roots under aluminum stress, with putrescine showing the most pronounced inhibitory effect (Figure 5e).

### 2.5. Expression Analysis of ALMT Gene Family Members Under Aluminum Stress with Exogenous Polyamines

**Figure 6:**
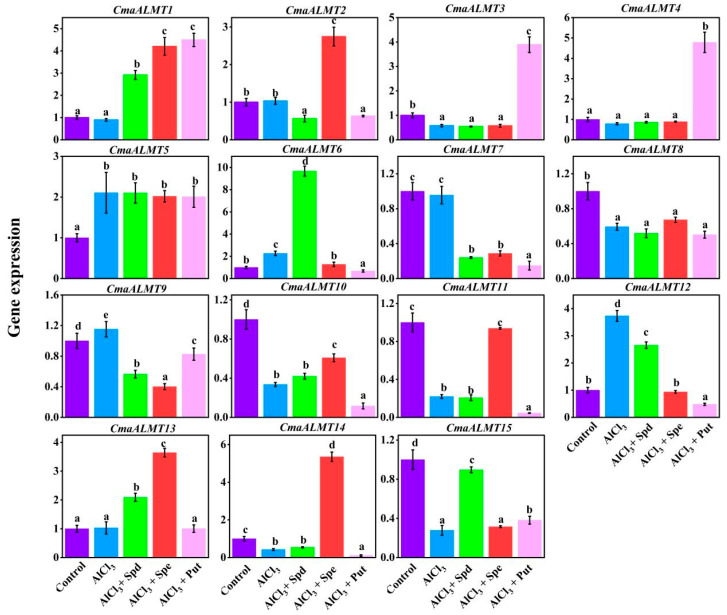
Effects of Exogenous Polyamines treatment on the Expression of *CmaALMT* Gene Family Members in Pumpkin Under Aluminum Stress. Each data point represents the mean ± S.E. (*n* = 3). Different lowercase letters on the bar indicated significant difference (*p* < 0.05).

RT-PCR analysis revealed distinct response patterns among the different *CmaALMT* gene family members when exposed to Al stress and exogenous polyamines (Figure 6). Put demonstrated significant synergistic transcriptional induction effects, whereby the expression levels of *CmaALMT1*, *CmaALMT3*, and *CmaALMT4* under Al stress were substantially higher than those in the control group without Put. These findings support the hypothesis that Put enhances root malate secretion by upregulating these genes. In contrast, Put notably inhibited Al-induced expression of *CmaALMT2* and *CmaALMT12*. Spd regulation exhibited marked specificity, with *CmaALMT6* showing strong induction and expression levels significantly exceeding those of the other treatment groups under Al stress. Additionally, Spd significantly enhanced the expression of *CmaALMT1*, *CmaALMT2*, and *CmaALMT13* under Al stress. Spm demonstrated a distinctive regulatory characteristic through its potent restorative effect on Al-suppressed genes; the expression levels of *CmaALMT11*, *CmaALMT14*, and *CmaALMT15* showed significant recovery following Spm treatment, occasionally surpassing levels observed under singular Al treatment. Moreover, Spm strongly induced *CmaALMT13* expression while maintaining elevated expression of *CmaALMT2*.

## 3. Discussion

Al toxicity in acidic soils constitutes a major abiotic stress that severely limits global crop productivity [1,2]. Plants have developed various detoxification mechanisms throughout evolution, with the root secretion of organic acids, such as malate, to chelate Al^3+^ representing a key external exclusion mechanism [12]. The ALMT family of proteins plays a central role in this process. This study identified 15 ALMT genes in the pumpkin genome, which is comparable to the numbers found in *Arabidopsis thaliana* (14 members) [47], tomato (16 members) [48], and melon (13 members), indicating functional conservation and importance across plant species. Phylogenetic analysis demonstrated that the 15 CmaALMT proteins could be classified into four evolutionary clades exhibiting clear functional differentiation, consistent with studies on species such as soybeans and potatoes [18,19]. Clade I, widely recognized as a crucial subgroup for Al-activated malate efflux, includes functionally validated members such as *AtALMT1* in *Arabidopsis* [17]. This clade contained five pumpkin *ALMT* members, with the subgroup size reflecting conservation of the core environmental adaptation function during pumpkin evolution. Among these, *CmaALMT6* was significantly induced under Al stress, with expression levels increasing proportionally with Al concentration, establishing it as the primary candidate gene for Al toxicity mitigation in acidic soil through Al-activated malate efflux, consistent with findings in wheat and rapeseed [15,16]. The ALMT members in Clade II primarily function as broad-spectrum anion channels that regulate stomatal movement and water homeostasis [49]. Two pumpkin members, *CmaALMT14* and *CmaALMT15*, belong to this clade and are likely expressed in guard cells and participate in water balance and stomatal conductance regulation, representing crucial molecular components for the environmental stress response of pumpkin. Clade III represents a basal and highly conserved subgroup within the *ALMT* phylogenetic tree, containing only *CmaALMT5* from pumpkin. High cross-species conservation and low redundancy suggest its fundamental importance in basic cellular physiology. Thus, pumpkin *CmaALMT5* likely carries the most conserved and essential ALMT family functions, such as maintaining intracellular ion homeostasis, metabolic connectivity, and basic developmental signaling. Beyond Clade I, this study revealed the multifunctionality of the ALMT family in Al stress response. For instance, Clade II member *CmaALMT12* was significantly upregulated under Al stress. While homologous genes in this clade (e.g., *AtALMT12* in *Arabidopsis*) function as anion channels in guard cells for stomatal movement regulation, Al-induced expression of *CmaALMT12* in roots suggests that Al stress may trigger a systemic response, utilizing water and ion homeostasis regulation for overall protection [50]. Conversely, *CmaALMT10* and *CmaALMT11* were continuously downregulated under Al stress, whereas *CmaALMT7* and *CmaALMT9* remained stable, reflecting functional divergence and regulatory complexity. This differential regulation likely stems from diverse cis-acting elements in their promoter regions. Previous analyses have revealed that the promoter regions of different *CmaALMT* genes contain various regulatory elements for hormone response, stress response, and transcription factor binding, forming a complex transcriptional regulatory network. Under Al stress, specific genes (such as *CmaALMT5*, *CmaALMT6*, and *CmaALMT12*) activate detoxification functions, whereas other genes may suppress or maintain optimization of resource allocation and sustain basic cellular physiological activities [51]. Clade IV represented the region of significant pumpkin *ALMT* family expansion and contained seven family members. The core function of this clade involves vacuolar membrane-localized malate transporters; for example, tomato *SlALMT9* directly influences fruit acidity, flavor, and commercial quality through vacuolar malate sequestration [48]. The predominance of Cucurbitaceae *ALMT* members in this clade potentially results from selection pressure for fruit organic acid accumulation and quality optimization during cucurbit crop domestication.While bioinformatic and gene expression analyses yield important clues about the roles of ALMT members, direct experimental approaches such as functional characterization and protein-protein interaction assays remain imperative to decipher their transcriptional regulation across different physiological contexts.

PAs are a class of low-molecular-weight, biologically active, nitrogen-containing aliphatic alkaloids widely distributed in plants. The most common PAs in higher plants include the diamine Put, the triamine Spd, and the tetra-amine Spm [23,24]. PAs possess multiple positive charges under physiologically acidic conditions, enabling them to bind strongly to negatively charged phospholipids on the cell membrane through hydrogen and ionic bonds, thereby protecting tissues [52]. The detrimental effects of Al^3+^ stress are most pronounced in root cells, inhibiting root growth and nutrient absorption, while simultaneously compromising cellular antioxidant and biomembrane systems [53]. Evidence suggests that polyamines may improve internal aluminum tolerance through a dual mechanism involving the enhancement of antioxidant defenses and the regulation of osmotic balance.Research involving saffron root tips exposed to various Al^3+^ concentrations demonstrated that polyamine application reduced Al^3+^ concentration within the root tips, enhanced cell viability, and decreased the synthesis and accumulation of MDA, thereby mitigating damage to the antioxidant enzyme system [54,55,56].In this study, it was found that polyamines enhanced internal aluminum tolerance in pumpkin by reducing proline content and relative electrical conductivity in the roots under aluminum stress. On the other hand, the study revealed that polyamines promoted the level of malate secreted by the roots under aluminum stress and decreased aluminum content in the root tips. This led to the hypothesis that root-secreted malate may form stable chelates with aluminum ions, altering aluminum availability and thereby reducing its uptake by the roots.

ALMT is widely recognized as an important membrane protein involved in Al^3+^-activated malate transport. In this study, it was observed that alongside the increased efflux of malate, polyamines exhibited distinct regulatory patterns on different members of the *ALMT* gene family in pumpkin.Spd treatment induced the expression of *CmaALMT6*, which was identified as a key candidate gene for Al-activated malate efflux in the initial stress experiments. This indicates that Spd serves a direct and efficient role in enhancing the external Al exclusion mechanism. While Put proved to be the most effective in elevating the overall malate content, its regulatory mechanism appears more intricate, as it coordinately and significantly induces the expression of *CmaALMT1*, *CmaALMT3*, and *CmaALMT4*. Additionally, Spm effectively restored the expression of genes suppressed by Al stress, including *CmaALMT11*, *CmaALMT14*, and *CmaALMT15*. Significantly, *CmaALMT1* was induced by all three polyamines and belonged to the core Al-tolerance Clade I along with *CmaALMT6*, suggesting its crucial role in the polyamine-mediated Al exclusion mechanism. This differentiated regulatory pattern indicates complex signal crosstalk between polyamines and Al stress response pathways. Similar beneficial effects of polyamines (particularly Put) on Al resistance have been documented in species such as rice [40]. By establishing connections between specific polyamines and the regulation of particular *ALMT* gene members in pumpkin, may lay the groundwork for further investigation into the mechanisms by which polyamines regulate aluminum stress. However, to refine the “polyamine-ALMT-malate” model for aluminum stress response, it remains necessary to identify the transcriptional regulators linking polyamines to ALMT and to verify the function of pumpkin ALMT in mediating malate transport under Al^3+^ stress. This study provides new clues for enhancing crop adaptation to acidic soils.

## 4. Materials and Methods

### 4.1. Materials and Stress Treatments

The study employed ‘Mini Beibei’ pumpkin (Cucurbita moschata ‘BeBe’) as the experimental material. Seeds were surface-sterilized with 75% ethanol for 30 s, followed by immersion in 5% sodium hypochlorite solution for 10 min and thorough rinsing with distilled water. The sterilized seeds were germinated on moist gauze in darkness at 28 °C for 48 h. The uniformly germinated seeds were transferred to Hoagland’s nutrient solution for hydroponic cultivation. Growth conditions were maintained at day/night temperatures of 25/22 °C, a photoperiod of 14/10 h (light/dark), 65% relative humidity, and light intensity of 300 μmol·m^−2^·s^−1^. The nutrient solution was replaced every three days. Seedlings were cultivated for 10 d until two true leaves developed before initiating Al stress and exogenous substance treatments.

The experimental protocol consisted of two treatments: Aluminum Concentration Gradient Treatment: Seedlings were cultivated in Hoagland’s nutrient solution (pH 4.5) containing 0, 250, 500, 750, and 1000 μmol·L^−1^ AlCl_3_, respectively, for two weeks. Exogenous Substance Alleviation Treatment: Under 1000 μmol·L^−1^ AlCl_3_ stress conditions, 0.1 mmol·L^−1^ of spermidine, spermine, or putrescine was independently administered for co-treatment over a two-week period. All exogenous substances were administered as either hydrochloride salts or free bases, and the pH of all treatment solutions was adjusted to 4.5. The control seedlings were maintained in Hoagland’s nutrient solution (pH 4.5) without AlCl_3_ supplementation.

### 4.2. Bioinformatic Analysis of the Pumpkin ALMT Gene Family Members

The genomic protein sequences of pumpkin (*Cucurbita moschata*) were obtained from the Cucurbit Genomics Database (CuGenDB; http://cucurbitgenomics.org/). The Hidden Markov Model (HMM profile, PF11744) for the ALMT protein family was acquired from the Pfam database, and the HMMER 3.3.2 software was employed to screen the local pumpkin protein database for candidate ALMT members. All candidate sequences were validated using the NCBI Conserved Domain Database (CDD) and SMART database to confirm the presence of complete ALMT domains. For phylogenetic analysis, the identified pumpkin CmaALMT protein sequences were subjected to multiple sequence alignment (ClustalW) with ALMT protein sequences from *Arabidopsis thaliana*, *Solanum lycopersicum*, and *Cucumis melon* retrieved from the Phytozome database. A phylogenetic tree was generated using the neighbor-joining method in MEGA 11.0 software, implementing 1000 bootstrap repetitions to evaluate branch confidence levels. The TBtools-II software facilitated the visualization of exon-intron structures for each *CmaALMT* gene based on the genome annotation file (GFF3). Conserved motifs of ALMT proteins were examined using the MEME Suite 5.0 online tool with parameters configured for a maximum of 10 motifs and widths between 6 and 50 amino acids. For promoter cis-acting element analysis, TBtools-II was used to extract 2000 bp sequences upstream of the transcriptional start site (TSS) for each *CmaALMT* gene, which were then analyzed using the PlantCARE database for element prediction and functional annotation.

### 4.3. RNA Extraction and Quantitative Real-Time PCR (qRT-PCR) Analysis

Total RNA was isolated from pumpkin root tip tissues using an RNA extraction kit (AikeRei Biotechnology Co., Ltd., Wuhan, China). The RNA quality assessment included concentration, purity, and integrity measurements using a NanoDrop 2000 spectrophotometer (Thermo Fisher Scientific, Waltham, MA, USA) and agarose gel electrophoresis. Qualified RNA samples were reverse transcribed to cDNA using a reverse transcription kit (AikeRei Biotechnology Co., Ltd.). Primer Premier software (version 5.0) was used to design specific primers for the identified *CmaALMT* genes (Table S1), with *CmaActin* as the internal reference gene. qPCR analysis was conducted using SYBR Green qPCR Premix (AikeRei Biotechnology Co., Ltd.) on a real-time fluorescence qPCR system. The thermal cycling conditions comprised initial denaturation at 95 °C for 30 s, followed by 40 cycles of 95 °C for 5 s and 60 °C for 30 s. Gene expression levels were quantified using the 2^−ΔΔ^Ct method.

### 4.4. Determination of Relative Electrolyte Leakage, Proline, Malate Content and Al Content

The proline and malate contents in the root tip tissues were quantified following the manufacturer’s protocol for the Proline Content Detection kit (Solarbio Science & Technology Co., Ltd., Beijing, China) and The relative electrolyte leakage was estimated as previously described [34]. After 14 days of cultivation, the malate content in the culture medium was measured for different treatments using a Malate Content Detection Kit (Solarbio Science & Technology Co., Ltd., Beijing, China) with a 4 mL aliquot. For aluminum content analysis, 10 treated melon root tips (14-day) were incubated in 1.2 mL of 2 mol/L HCl and shaken for 24 h. Subsequently, 1 mL of the supernatant was diluted with 3 mL of water (resulting in a final acidity of 0.5 N) and analyzed by inductively coupled plasma optical emission spectrometry (ICP-OES) [57].

### 4.5. Statistical Analysis

Each experiment included a minimum of three biological replicates. Data processing was performed in Excel, followed by One-way Analysis of Variance (ANOVA) using SPSS 26.0. Duncan’s multiple range test (*p* < 0.05) was used to determine significant differences between groups. Origin 2021 software was used for figure generation and correlation visualization analyses.

## 5. Conclusions

This study successfully identified 15 *CmaALMT* genes in the pumpkin genome and categorized them into four evolutionary clades (Clades I-IV) through phylogenetic analysis, indicating significant functional differentiation among family members. *ALMT* genes in pumpkin show differential expression under aluminum stress. PAs alleviate aluminum toxicity symptoms and these effects correlate with internal malate accumulation. The regulation of pumpkin ALMT family members by exogenous polyamines exhibited distinct specificity patterns. Spd demonstrated a specific synergistic induction effect, maximizing the expression of the core Al tolerance gene *CmaALMT6*. Spm effectively restored the expression of Al-suppressed genes, including *CmaALMT11*, *CmaALMT14*, and *CmaALMT15*. Spm also substantially induced *CmaALMT13* expression while maintaining elevated expression of *CmaALMT2*. Put exhibited strong synergistic transcriptional induction, elevating the expression of *CmaALMT1*, *CmaALMT3*, and *CmaALMT4* to peak levels. However, whether polyamines enhance plant aluminum tolerance by regulating ALMT transcription remains to be supported by more evidence. Current studies have not identified key transcription factors in the polyamine signaling pathway that regulate ALMT. Therefore, further efforts should be made to refine the establishment of this regulatory model.

## Figures and Tables

**Table 1:** Molecular characteristics of CmaALMT genes in pumpkin.

Gene ID	Gene Name	N-amino Acids	Molecular Weight	Theoretical pI	Transmembrane Domain	Subcellular Localization
CmaCh02G014150	*Cma* *ALMT1*	539	49624.01	8.03	6	Plas
CmaCh04G015300	*Cma* *ALMT2*	570	64330.74	6.56	5	Plas
CmaCh06G005250	*Cma* *ALMT3*	451	49427.12	6.89	6	Plas
CmaCh07G008230	*Cma* *ALMT4*	473	52024.12	6.24	6	Plas
CmaCh08G001080	*Cma* *ALMT5*	478	52692.11	5.84	7	Plas
CmaCh10G002420	*Cma* *ALMT6*	570	64065.79	6.49	6	Plas
CmaCh10G008660	*Cma* *ALMT7*	915	100938.32	8.31	11	Nucl
CmaCh10G008670	*Cma* *ALMT8*	328	36890.29	9.26	6	Plas
CmaCh11G002660	*Cma* *ALMT9*	576	64695.56	6.17	6	Plas
CmaCh11G009010	*Cma* *ALMT10*	712	77716.19	8.46	5	Plas
CmaCh11G009020	*Cma* *ALMT11*	449	49716.03	8.56	6	Plas
CmaCh14G003580	*Cma* *ALMT12*	467	51243.36	8.35	6	Plas
CmaCh18G010330	*Cma* *ALMT13*	1215	136721.87	6.56	5	Plas
CmaCh19G000480	*Cma* *ALMT14*	436	48169.09	8.61	5	Plas
CmaCh19G004590	*Cma* *ALMT1* *5*	519	57705.82	8.99	5	Plas

## Data Availability

The original contributions presented in the study are included in the article/Supplementary Materials; further inquiries can be directed to the corresponding author.

## Supplementary Materials

The following supporting information can be downloaded at: https://www.mdpi.com/article/10.3390/plants14243745/s1, Table S1: Molecular characteristics of CmaALMT genes in pumpkin. Table S2. Primers used in this Cmaudy. Table S3. Amino acid sequences of ALMTs in pumpkin.
