# Supplementary material for: Expression Profiling of the Aluminum-Activated Malate Transporter (ALMT) Gene Family in Pumpkin in Response to Aluminum Stress and Exogenous Polyamines
Source: Plants (Basel). 2025 Dec 9;14(24):3745. doi: 10.3390/plants14243745 (PMC12736512; doi:10.3390/plants14243745)
Supplement: Supplementary file 1 [file plants-14-03745-s001.zip › Table S1.pdf]

Table S1 Molecular characteristics of CmaALMT genes in pumpkin.

Gene ID	Gene name	N-amino acids	Molecular weight	Theoretical pI	Transmembrane domain	Subcellular localization
CmaCh02G014150	*CmaALMT*1	539	49624.01	8.03	6	Plas
CmaCh04G015300	*CmaALMT*2	570	64330.74	6.56	5	Plas
CmaCh06G005250	*CmaALMT*3	451	49427.12	6.89	6	Plas
CmaCh07G008230	*CmaALMT*4	473	52024.12	6.24	6	Plas
CmaCh08G001080	*CmaALMT*5	478	52692.11	5.84	7	Plas
CmaCh10G002420	*CmaALMT*6	570	64065.79	6.49	6	Plas
CmaCh10G008660	*CmaALMT*7	915	100938.32	8.31	11	Nucl
CmaCh10G008670	*CmaALMT*8	328	36890.29	9.26	6	Plas
CmaCh11G002660	*CmaALMT*9	576	64695.56	6.17	6	Plas
CmaCh11G009010	*CmaALMT*10	712	77716.19	8.46	5	Plas
CmaCh11G009020	*CmaALMT*11	449	49716.03	8.56	6	Plas
CmaCh14G003580	*CmaALMT*12	467	51243.36	8.35	6	Plas
CmaCh18G010330	*CmaALMT*13	1215	136721.87	6.56	5	Plas
CmaCh19G000480	*CmaALMT*14	436	48169.09	8.61	5	Plas
CmaCh19G004590	*CmaALMT*15	519	57705.82	8.99	5	Plas
