# Supplementary material for: Expression Profiling of the Aluminum-Activated Malate Transporter (ALMT) Gene Family in Pumpkin in Response to Aluminum Stress and Exogenous Polyamines
Source: Plants (Basel). 2025 Dec 9;14(24):3745. doi: 10.3390/plants14243745 (PMC12736512; doi:10.3390/plants14243745)
Supplement: Supplementary file 1 [file plants-14-03745-s001.zip › Table S2.pdf]

Table S2. Primers used in this Cmaudy

Primers	Sequence 5′-3′	Purpose
Cma-Actin-F	AGCCATCTCTCATCGGTAT	
Cma-Actin-R	CATGGTTGAACCACCACTG	
Cma- ALMT1-F	CTTGGAAGCTTGCTTGCAGG	
Cma- ALMT1-R	CAGTTCTCAGTGGGTTCCCC	
Cma- ALMT2-F	CCGCAAATTCTACTTCGCCG	
Cma- ALMT2-R	TGTCCCCAATGCACGATTGA	
Cma- ALMT3-F	GTTTCGGTTTCGGCGTATCG	
Cma- ALMT3-R	CGTTTCACCAGCCCAAACAG	
Cma- ALMT4-F	TCGATGGGGAGTGTGGAGAT	
Cma- ALMT4-R	ACCATCTCCCCATTGCTTGG	
Cma- ALMT5-F	AGCTTGCTGATGGCTTGGAT	
Cma- ALMT5-R	CCATGTGCTGGTTCCCATCT	
Cma- ALMT6-F	TGACCCTAGGCCATCAGACA	
Cma- ALMT6-R	GCGCTCGCATTTTCGTATGT	
Cma- ALMT7-F	AAGTCGAAGGCAGCGATGAA	
Cma- ALMT7-R	TTCACCAACGGACTCTGCAA	RT-qPCR
Cma- ALMT8-F	ATGACGAAGAAGCTGGCGAA	
Cma- ALMT8-R	ACCTCGTCCTAGTGTCCCTC	
Cma- ALMT9-F	AGGAATTCCCCGACCTAGCT	
Cma- ALMT9-R	AAGCATCAAAGCGAGACCCA	
Cma- ALMT10-F	GGGAAAGACGACCCCAGAAG	
Cma- ALMT10-R	CTCGAAGACGACTACGACGG	
Cma- ALMT11-F	GAGGTTCGTTCCGAGCATCA	
Cma- ALMT11-R	CACAAACGCAGACACCAAGG	
Cma- ALMT12-F	CACCCTCATCCCAAGTGCAT	
Cma- ALMT12-R	CACTGCCTCCGACAAGTCTT	
Cma- ALMT13-F	ACTCAATCCGTGATGCAGCA	
Cma- ALMT13-R	ACCGCACGAAGAACAGTCAT	
Cma- ALMT14-F	TATTGCGACGTACTTGCGGT	
Cma- ALMT14-R	TCGACAAGCGTTCTCTTGCT	
Cma- ALMT15-F	CGATAAAATCGCAGCCACGG	
Cma- ALMT15-R	AGTCGGTTTTCACGCTCGAT	
