# Supplementary material for: Expression Profiling of the Aluminum-Activated Malate Transporter (ALMT) Gene Family in Pumpkin in Response to Aluminum Stress and Exogenous Polyamines
Source: Plants (Basel). 2025 Dec 9;14(24):3745. doi: 10.3390/plants14243745 (PMC12736512; doi:10.3390/plants14243745)
Supplement: Supplementary file 1 [file plants-14-03745-s001.zip › Table S3.pdf]

Table S3. Amino acid sequences of ALMTs in pumpkin

Gene ID and Name	Amino acid sequences
CmaCh02G014150<br>(CmaALMT1)	MNGKTGNTVIDIDFSTKPEPIGAKAENLAASCKAPIGSVLGFCKEDSN<br>RVLFSLKVGLAVVLVSLLILFRAPYDVFGTNIIWAIITVAIMFEYTVGAT<br>FNRGFNRALGSLLAGILAIAVAQLALRTGPIAEPIIIGISIFLVGSVTSFMK<br>LWPSLVPYEYGFRVLLFTYCLIVVSGYRMGNPLRTAMDRLYSIAIGGF<br>VAVLVNVLVFPIWAGEQLHKDLVATFNSLADSLQECVDKYLEDEGSVH<br>NIGQQLPKAVMDEFPEEPAYKKCKATLNSSAKFEALATSAKWEPPHGR<br>FRHFFYPWTEYVKVGAVLRYCAYEIMALHGVLHSRIQAPYKLRITFKS<br>EIQETANQAAELLRSLGKDINNMQQSLKIYLLKNVHTAADKLQQALH<br>THSYPLTPTCASLHLSSKLQSKLSTASSNSLYDLPSLLAELNGNGPERS<br>LNQQNSHGVAAGTQAEAYVEFVRKQSRRQNSWPLREMDVFDDDGG<br>VPVEFLQRMRKLESTAATSLANFTSLLIEFVARLDYLVETVDELSKMA<br>KFKDIIIS
CmaCh04G015300<br>(CmaALMT2)	MTGKYGSIRQSFLDQNREQLLSRKGHSDFGLHGNDDAGADDDAKCR<br>CFCTVSDAITNFWKGLHDTTVKLCDMSRSDPRKFYFAAKMGLSLALT<br>SLLIFIREPLKEVAQYSIWAILTVVVVFEFSVGATLNKGFNRALGTLSA<br>GGLALGIAEISVSAGEFEEVAIVISIFLAGFFASYCKLYPPMKTYEYGFR<br>VFLLTFCIVLVSGSSSSFFHTAFYRLLHIAVGAGMCLVVNICIYPIWAGE<br>DLHKLVVKNFKNVSSSLEGVVSEYLQCVEYERVSSKILTYQASDNPIY<br>NAYRSAVQSSSQEDTLLDFARWEPPHGPYKTFKYPWHNYVKVSGALR<br>HCAFMVMAMHGCILSEIQAPPEKRKVFAKELQRVGTEGAKFLRALGS<br>KVEKMEKLSSGDMLFDVHDAAEALQIKIDERSNMLVNSDRWRTGKQ<br>HREQEEPQHFIDVKDDHNKQLVIDSLNETMYAQHSSIGVHPPMTEWV<br>STDNVFNKHLVSWPRLSFLMDTVPNERESKVHESASSLSLATFASLLIE<br>FVARLQNLRNAFEELNRVFLFLWSLSLSFFLSDDRFLKLSQPGH
CmaCh06G005250<br>(CmaALMT3)	ISCKVLAMEILKNKAMEVAMKVKKLGQDDPRRIIHSIKVGVALTLVSL<br>FYYWRPLYDGFGASGIWAVITVVVIFEFTVGATLSKGFNRGLGTLLAG<br>GLGVGVDYLANLSGQKGEPFVLGVFVFLIAASATFSRFFPGIKARYDY<br>GVLIFILTFSMVSVSAYRFDEFLMMAYQRLSTIVAGGAICIIICIVVCPV<br>WAGETLHNLVTSNLDKLGDYLEGFGGEYFYCCDDEEQANFARWEPK<br>HGNFSFRHPWKHYLKIGSYARQCAYHIQVLNTHLNFQPPNLSQFRTIIQ<br>VQCNTLSSESGKALKALATAMKTMSDPSPTSQVHLNAAKSAINDLKN<br>CLKSATTQTPDECSNLLAIIPDATVASILIEIVKSVEDLCEAVGELSVKA<br>HFKSVEGSVAVEKPQLLHRGAIKPFVEAEDEGENGHVVITVREIDNED<br>SPQKKGSSDTNGLVNKK
CmaCh07G008230	MLAMAADQPKLGAMSVMERIVKLGRDDPRRIIHSLKVGLALTFVSLL

(CmaALMT4)	YYWRPLYDGFGIAAIWAVLTVVVIFEFTVGATLSKGLNRGLGTLLAGA<br>LGVGAQHFASLFGQIGEPIVLGFFVFLLAAASTFSRFFPRIKARYDYGV<br>LIFILTFSLVSVSGYRVEKILELAHQRLSTILIGGATCIFISLFICPVWAGE<br>SLHNIIVSNIEKLANYLEGFGGEYFRYEDRENVDEDSTHNKSSSLQAY<br>KTVLTSQSSEESLANFASWEPRHGKFSFRHPWKQYLKIGSLARQCAYQ<br>IESLNGYINVPTDIQVAIQFRRRIEESCNAISSESGKALRILASSIKTMTN<br>PSLSSKTHIENATAAIDELKNTLKTGYMESSDLLGIIPDATVCCILIDIVK<br>SVEKILEAIEELGVLASFKSVEPTVSPEKSTQLLHRGIVNPLFDGECGD<br>HVVIMVDNEVEVGKSQGLEGKPSNGEMVCK
CmaCh08G001080<br>(CmaALMT5)	MASGGSLEWRVNVGDGATEVLRPEGSFVSRMVMGVKGFVWGLFMK<br>GWMFLEKAWGIANSEPKKAVHGLKVGLALSIVSVFYYMRPLYEGIGG<br>NAMWAIMTVVVTFESSVGATFYKCVNRVIGTSLAGGLGIGVHWVAA<br>ESGDRFEPIILGISLFLLASATTFSRFIPSIKTKFDYGAMIFILTFCLVSISS<br>YRVDKLFELAHTRLTTIAIGTSLCIIISMLFCPMWAGSQLHSLVFRNLD<br>KLADGLDGCVSEYFKNVDDPEMENEDQECHDSKAQSYKCVLNSKAS<br>EESMANFARWEPAHGRFGFRHPWKQYLEVGVAMRKCAYCIEALHDC<br>LNSEIKAPNSLKLLLVEPCKALSTSSSKVLKELSIVIKTMKKSTKIDLEV<br>SDMNIAVQELQAAIASFPSTQTANEEHEAATIPPLMELLPLATLVSLLIE<br>TASRIEHVVNAVETLANVANFDSEEEKKRSCLDNNDHVAMRVFPEA
CmaCh10G002420<br>(CmaALMT6)	MAAKYGSFKQSFAEKRERLLSAKEFPDLIYGTLQDNPSFCSSFRSVPY<br>RISNLWNSVQDVLCKSWQMGVSDPRKIVFSAKMALALTLISLLIFFKQ<br>PVEELSRYSVWAILTVVVVFEFSIGATLSKGLNRGLGTLSAAGLALGM<br>AGLSVLAGEWEELVVAISIFITGFFATYAKLYPTMKPYEYGFRVFLLTY<br>CFIMVSGYRTRDFIHTAITRFLLIALGAGVCLVVNICIYPIWAGEDLHNL<br>VVKNFAGVAASLEGCVDSYLNCVEYERIPSKILTYQASDDPLYKGYRS<br>AMESMSQEESLMGFAIWEPPHGRYKMLKYPWKSYVEVAGALRHCAF<br>AIMGLHGSILSEIQAPAERRHVFRNELRRVSYEGAKVLHELGNKLKK<br>METLDCAAILSEVHDAAEELQKKIDAKSYLLVNSESWEIGNHHDDNV<br>GRSQELLNMDDEETRFHEYRSLSEAVLDLRAFPVPKTWNDPRPSDINS<br>VQAAAFPPTNKMFKKLGSWPAQVSNKPNGVIHAEESKTYENASALSL<br>ATFTSLLIEFVARLQNVVDSFEELSKKAKFKDPMERETSKAPEC
CmaCh10G008660<br>(CmaALMT7)	MEMGSDEKVGLMSGRFVWVKGLFAKLVEVANKTRALGKDDPRRVIH<br>SLKLGLTLTIVSMLYYYKPLFANFGVSAMWAVITVVVVFEFSVGATLG<br>KGLNRAFATLFAGALGAGAHHLAALSGHVGQPIITSIFVFLLACILTFM<br>RFFPSIKAKYDYGMMIGILTFSFVSISGVGDDEILLLLQKRVSTIFLGVC<br>VCVVISIFISPFWAGQDLHNRIALNIENLALFFEGYGSVEYFKTLQDRE<br>AKDDKSIQAYKSILKSSGIEDTLYNFARWEPGHGCFQFRHPWKQYLKI<br>GALTYQCAFRIDALHRNLSSSSFQVSQEIQTQIQEPCMEMSMETGKAL

	RQLVSSIREMTQPTLAEIHTNNSKAAAKKLKASLKSSRLWENCDLLTL<br>IPAANVGTLLVDVVDITEKIAEAVQELASLAHFKAAKPADSAIQSEKEK<br>LEPNIGALVGLSFQVGVNGNFGSYAALAVEMSDEKNEGVLARWGEG<br>LKAKSSSLKAKVVELLRKTKKLAKDDPRRIVHSFKVGLAITLVSLFYY<br>FEPLYDGLGASAMWAVLTVVVVFEFSVGATLGRGLNRVLATSLAASL<br>GFGAHFLADLAGDKAQPVILSLSVFIFAAITTFIRFFPRIKARYDYGFLIF<br>ILTFCLVSVSGYRDDEILQVAYRRALTILLGTFIAILICLFICPVWAGDDL<br>HSLVSNNIQLLANFLQGFGVQYSNESKEDELLEGCKSVLTSRQTEESL<br>VNFARWEPRHGAFKFRHPWKQYRKIGSLTRQSAYRLESLNSYLVTET<br>QTPLHIRDRLMKSCSKMSIESSNALEDIASDLRTMTQPTFPNPHIEKSK<br>AAMKDLKAALKIGPGDGDIDLLEIVPIATVASLLIDTISCIEKIAESVGE<br>LGSLANFKCVEAGKSSGLELEQEHQKLPNSAIVNDHCHVVIVD
CmaCh10G008670<br>(CmaALMT8)	MATEKSSNEYEGLLPFCLRWVKPIFTKLPAMAVELATMTKKLAKDDT<br>RRVVHALKVGLAITLVSLFYYFKPLYDGFGTSTIWAVVTVVVVFEFSV<br>GGTLGRGLNRVMATLLAGALGFGAHYLASLGGDTGRPIMLGLFVFVL<br>AAISTFIRFFPKMKARYDYGLLIFILTFCMVSLSGYRDDEIAKLAYSRM<br>LTILIGCCFTVFVCVFVCPVWAGTDLHHLVASNIDSLATFFHGFGAEYF<br>GLLQEQGEVGKVDMQKYRTVLNSKSNEESLTNLARWEPRHGKFRYR<br>HPWKVYLEIGSLNRECACRLEVLNGYLQTKRTQVRFDASFAR
CmaCh11G002660<br>(CmaALMT9)	MVAKYGSFKHSFAERRERLLSAKEFPDLAFGAFQTIQENPSSCSPFLGI<br>SRLWKSAKDVVYEAWQMGVSDPRKIVFSAKMGLALMLISLLIFFKQP<br>VEELSRYSVWAILTVVVVFEFSIGATLSKGFNRGLGTLSAGGLALGMA<br>ELSVLAGDWEEVVVVISIFITGFFATYAKLYPTMKPYEYGFRVFLLTYC<br>FIMVSGYRTREFIHTAVTRFLLIALGAGVCLVVNICIYPIWAGEDLHNLV<br>VKNFAGVAASLEGCVDNYLNCVEYERIPSKILTYQASDDPLYKGYRSA<br>VESMSQEESLMGFAIWEPPHGRYKILKYPWKNYVQVAGALRHCAFAV<br>MALHGCILSEIQAPAERRQVFGNELRRVGYEGAKVLRELGNKLKRME<br>TLDSASILSEVHDAAEELQKKIDAKSYLLVNSESWEIGNRSEDVGQPQ<br>ELLNLDDEETRFREYRSLSEAVLDLRAFPVPKSSDYTVSSDMNSIHSAA<br>VPPTKMFKKLGSWPAQISVKPNGVIQEESKTYENASALSLATFTSLLIE<br>FVARLQNLVDSFDELSAKATFNDPVEWENLKSPGFWRRFCNCFKV
CmaCh11G009010<br>(CmaALMT10)	MVEGSKETLTNDHHGWLKAKAWKLHKKMVELLNNATKVGKDDPR<br>RIVHSLKLGLAITTVSLFFYFEPLYDGLGASSIWAIITVVVVFEFSVGAT<br>LGKGMNRAIATLIAGAIGFGAHYITSFTGNIWHPILLGILIYTISATASYC<br>RFFPKLKAKYDYGLLIFILTFNMVALSGFRDDEILKLAWHRLANILVG<br>AFIAVAICIFVRPVWAGADLHQFVATNIENLGTFLEGFGIEYFGACQGE<br>MVGGGNVMKYRSVLCSKQNEEALKIGTLSRDCAYCFEILNLYLNTDQ<br>IQLPLEIQRQYQEQCVQVCIESSKALKAMAKALRDIVPPALAKSHIEIA

KKTAGELKLLLASSHFHGDMNMVSTTTLILLLIDSFSCVEKIVDSIHEL
VSLARLNTALQKSDVTPTEQKGSGDKASSQPHHIVTIAQSQFADLLLC
NKAMATQALVSSSSLTSSVGAARQRLGPKPSLGSSRKAASFVVRAEAT
PPVKQGANRQLWFASKQSLTYLDGSLPGDYGFDPLGLSDPEGTGGFIE
PKWLAYGEVINGRFAMLGAAGAIAPEIFGSLGLIPPETALPWFKTGVIP
PAGTYNYWADPYTLFVLEMALMGFAEHRRFQDWYNPGSMGKQYFL
GLEKFLGGSGDPAYPGGPLFNPLGFGKDEKSMKELKLKEIKNGRLAM
LAILGYFIQGLVTGVGPYQNLLDHLSDPVNNNVLTSLKFH

CmaCh11G009020
(CmaALMT11)

MEMGSDEKVGLMRKGFEWVKKWVKGLVGKLVEVRSKATALGKDD
PRRVIHSLKLGLALTIVSILYYYNPLYANFGVSAMWAVMTVVVVFEFS
VGATLRKGLNRTFATLFAGALGAGAHYLAALSGHVGQPIITSIFVFLLA
CILTFMRFVPSIKAKYDYGMMIGILTFALVSISGIQDDEVVLLFEKRISTI
FLGVCVCVLISISISPVWAGQDLHNRIALNIENLAIFLEGYGSVECLKSL
QDEQFTQEYKSILKSSGIEETLYNSARWEPGHGCFQFRHPWNQYLKIG
ALTSQCAFRIDALCRNLSSSNIQVSQKIQTEIQETCMEMSMESGKALR
QLVSSIRELTQPTRAQIYTHNSKSAAKRLKTSLRSSHLWEDCDFLTLIPA
ATVGLLLVDVVECTEKISEAVQELASLAHFKSGKAEPMQSEKEKVQP
NIGVAFVTIPISSV

CmaCh14G003580
(CmaALMT12)

MGIEAVKNKAMEIAMKIKKLGQDDPRRIIHSIKVGVALTLVSLFYYWK
PLYDGFGASGIWAVMTVVVIFEFTVGATLSKGLNRGLGTLLAGSLGVG
VNYLANLSGQKGEPFVLGIFVFLIAASATFSRFFPGIKARYDYGVLIFIL
TFSLVSISGYRMDEFLTLAHQRLSTILVGGAICIMISILVFPVWAGETLH
NSVTSNINKLADYLEGFGGEYFYCCEDGEHGVATEKDKSFVEGYKAV
LNSKSMEDSMANFARWEPKHGKFRFRHPWKHYLKIGSLTRHCAYQIE
ALNRHLCPQLQTQDPSQLRRMIEVPCKTLSSESGKALKALATGMKAM
TNPSPSSQVHLNAAKSAMNDLKDSLKLTTAQTTNLLAIIPDATVASILIE
IVKSVEDLSEAVVELSLKAHFKSIEATVSPEKPQLLHKGTINPFVEAED
EQPHVVIRVRGTEDEDLTEKKGSSGTTSLPT

CmaCh18G010330
(CmaALMT13)

MAGKYGSIRQSFLDQNREKAFSRKGYSDFGLNSYDGAGDNVKCRCF
RTLTDSITNFLKGLRDTTVKLYEMGRSDPRKIFFAAKMGLSLAFVSLVI
FFREALKEVGQYTIWAILTVVVVFEFSVGATLSKGFNRALGTFSAGGL
AIGIAELSVLAGPFKEVIIVISIFLAGFFASYCKLYPPMKSYEYGFRLDFA
CWEPPHGPYKTFNYPWYNYVRVSGALRHCAFMVMAMHGCILSEIQA
PPEKRKVFAKELQRVGTEGAKFLRELGSKVEKMEKLSSVDMLLDVH
DAAEALQMKIDEKSDILVNSANWRPGKQHKEHEDLQHLIDTKDDHG
RQLVIESLNDTLDAQHSSIGFNPSITEWVSTESVFNKHLVSWPRLSFLN
DTVPNERESKVYESASSLSLATFTSLLIEFVARLQNLLNAFEELSEKAN
FKSPAEIKIEKEKVGCDVKEVDCAVRLRCAAAAAALRVLNILLHAQA

	CQSDLISIAVRAAFCLIWISLLYFVVDRKGIRSYGALILSTCILHVRKAR<br>VYRKRFYRKSLYKMSMVHEPLYPIAVLIDELKNDDIQLRLNSIHRLSTI<br>ARALGEERTRKELIPFLSENNDDDDEVLLAMAEELGVFIPYVGGVEH<br>AHVLLPPLETLCTVEETCVRDKAVESLCRIGSQMRESDLVDWFIPLVK<br>RLAAGEWFTARVSACGLFHIAYPSAPEMLKTELRSMYGQLCQDDMP<br>MVRRSAATNLGKFAATIEPAHLKTDTMTIFEDLTQDDQDSVRLLAVEG<br>CAALGKLLEPQDCVSHILPVMVNFSQDKSWRVRYMVANQLYELCEA<br>VGPEPTRTDLVPAYVRLLRDNEAEVRIAAAGKVTKFCRILSPELAIQHI<br>LPCVKELSSDSSQHVRSALASVIMGMAPVLGKVIHPSTRGIDGLTSLP<br>VLFNDATIEQLLPIFLSLLKDEFPDVRLNIISKLDQVNQVIGIDLLSQSLL<br>PAIVELAEDRHWRVRLAIIEYIPLLASQLGVGFFDDKLGALCMQWLQD<br>KILVLWTRIPKFYRGGYPYPSRRAMEKCLVYIWGQKANFGCFFDGDL<br>VYSIRDAAANNLKRLAEEFGPEWAMQHIVPQVLDMINNPHYLYRMT<br>VLRAVSLLAPVMGSEITCTKLLPVVISSSKDRVPNIKFNVAKVLQSLIPI<br>VDQPITLAMQVVEKTIRPSLVELSEDPDVDVRFFANQALQSIDHNLKR<br>MANSFMNHIIDQLTLSM
CmaCh19G000480<br>CmaALMT14	MGSAVVVSIPEEIQDQRVTDDVPVDQNKNKNKNKTAAVNKFRNLFDS<br>TRDRIKKQDMKKIFHSIKLGIALVVVSLLYLLNPLYKQVGDNAMWAI<br>MTVIVVFEFYAGATLSKGLNRGLGTIFGGGLGCFAGSFAQDVGGVAC<br>ASIIGVSVFVFGGIATYLRLIPSIKKKYDYGVMISILTFNLVVVSGMREE<br>KIMALARERLSTIAMGFAVCIFINLLIFPAWASDELHHSTVQSFHNLAN<br>SIQGCMEDYFSSADDKKKNKSDASFSSCKAILNTKSKEESLANFAKW<br>EPWHGKFGLNYPWNRYLQIGEVLRELAATVLSIKGCLQSPRQPTSGM<br>REAIKEACEMGGSSIAWALKELGEGIKNMKRCQIEGVIVPKLKLVRQE<br>MSGLITPSKLGVIENGDELAMASFVFLIMEILEKVEEVAREVEELEEAG<br>RFRTT
CmaCh19G004590<br>(CmaALMT15)	MQVNKHIQVTMDKIQHFPVLCWKTSKKVGAEDPRRIIHSLKVGLSLT<br>LVSLLYLIEPLFKRIGSNATWAVMTVVVVLEFTAGATLCKGLNRGLGT<br>LLAGSLAFFIEGVANRSGRVFRACFIGAAVFLIGAAATYMRFFPKIKKN<br>YDYGVVIFLLTFNLITVSSYRVDNVLKIAHDRLYTIAIGCGVCLLMSLL<br>IFPNWSGEELHNSTVLKLEGLAKSIEGCVNEYFFDTEIDDNKESCSGD<br>QIYKGYKAVLDSKSIDESLALQASWEPRHSSHCYRIPWQQYVKLGGV<br>LRHFGYTVVALHGCLQTEIQTPRSVRILFKDPCTRVAREVSKALIELAN<br>SIRNRRHCSPEILSDHLHEALQDLNKAIKSQPRLFLGSNRNKASNMLA<br>LAAAEAGQKRCRSGGSLSSVKTDSSALMEWKTKRASVQSREAERKV<br>LRPQLSKIAITSLEFSEALPFAAFASLLVETVAKLDNIIDEVEELGRIACF<br>KEFINGDDQEQQHITVKCEKPNINVSQNQLSSVSGAE
